# Supplementary material for: Short-term alterations in dietary amino acids override host genetic susceptibility and reveal mechanisms of Salmonella Typhimurium small intestine colonization
Source: bioRxiv. 2025 Mar 25:2025.03.25.645332. Preprint. [Version 1] doi: 10.1101/2025.03.25.645332 (PMC11974825; doi:10.1101/2025.03.25.645332)
Supplement: Supplement 1 [file media-1.pdf]

SUPPLEMENTARY MATERIALS

Table S1. Diet formulation of CA (D22011804) and AA diets (A20073101).

	**CA Diet** (Cat#:D22011894**)** *Matching Macronutrient Levels to 5L0D*	**AA Diet** (Cat#:A20073101) *Matching Macronutrient Levels to 5L0D*
**Ingredients**	**grams**	
Casein	280	0
L-Cystine	4.5	6
L-Isoleucine	0	10.6
L-Leucine	0	22.2
L-Lysine	0	18.5
L-Methionine	0	7.2
L-Phenylalanine	0	11.8
L-Threonine	0	10
L-Tryptophan	0	3
L-Valine	0	13
L-Histidine	0	6.4
L-Alanine	0	7.1
L-Arginine	0	0
L-Aspartic acid	0	17
L-Glutamic acid	0	53.5
Glycine	0	4.2
L-Proline	0	25
L-Serine	0	14
L-Tyrosine	0	13
**Total L-Amino aicds**	**4.5**	**242.5**
Corn Starch	312	312
Maltodextrin 10	110	110
Dextrose	150	150
Cellulose, BW200	100	100
Inuline	46	46
Soybean Oil	61	61
Mineral Mix S10026	10	10
DiCalcium Phosphate	13	13
Calcium Carbonate	5.5	5.5
Potassium Citrate , 1 H2O	16.5	16.5
Sodium Bicarbonate	7.5	7.5
Vitamin Mix V1001	10	10
Choline Bitartrate	2	2
FD&C Yellow Dye #5	0.05	0
FD&C Red Dye #40	0	0.05
**Total**	**1128.05**	**1086.05**
	**grams**	
**Protein**	251	243
**Carbohydrate**	599	599
**Fat**	61	61

**Fiber**	146	146
	**grams (%)**	
**Protein**	22.2	22.3
**Carbohydrate**	53.1	55.2
**Fat**	5.4	5.6
**Fiber**	12.9	13.4
	**kCal**	
**Protein**	1004	970
**Carbohydrate**	2397	2397
**Fat**	549	549
**Fiber**	3950	3916
	**kCal (%)**	
**Protein**	25	25
**Carbohydrate**	61	61
**Fat**	14	14
**Fiber**	100	100
**kCal/gram**	**3.5**	**3.6**

Table S2. Criteria for blinded scoring of histopathological changes.

Score	Submucosal edema	Epithelial damage	Exudate	Submucosal neutrophil infiltration (cells/high power field)	Submucosal mononuclear cell infiltration (cells/high power field)
0	No changes	No changes	No changes	No changes (0-5)	No changes (0-5)
1	Detectable (<10%)	Desquamation	Slight focal accumulation	(6-20)	(5-10)
2	Mild (10-20%)	Mild erosion	Mild focal accumulation	(21-60)	(10-20)
3	Moderate (20-40%)	Marked erosion and/or mild ulceration	Moderate multifocal accumulation	(61-100)	(20-40)
4	Marked (>40%)	Multifocal ulceration	Marked diffuse accumulation	(>100)	(>40)

Table S3. Primers used to construct plasmid or bacterial strains.

Name	Sequence
zraP_IR715_F1_ERV	CTAGAGGTACCGCATGCGATctcaatatttacagcaaacc
zraP_IR715_R1_ERV	tcgcgctaattgccctctctggttatcatcgcggcggcgg
zraP_IR715_F2_ERV	ccgccgccgcgatgataaccagagagggcaattagcgcga
zraP_IR715_R2_ERV	AATTCCCGGGAGAGCTCGATcatacctactcgcgtgccgg
yfiA_IR715_F1_ERV	CTAGAGGTACCGCATGCGATtactataccgcgcgcggcgc
yfiA_IR715_R1_ERV	ctggcatctttcaccgatgctgccggagtaatttccattt
yfiA_IR715_F2_ERV	aaatggaaattactccggcagcatcggtgaaagatgccag
yfiA_IR715_R2_ERV	AATTCCCGGGAGAGCTCGATgagatggtgggctttaccga
K1	CAGTCATAGCCGAATAGCCT
FrwC_del_Fwd	TGC CTG GGT GGG TAA CAG CTT CGG CGC GGG TTT TTT CGG G TG TAG GCT GGA GCT GCT TCG
FrwC_del_Rev	AAT TAA ACC GCC CCA ACC GGC ATA ACA CTG CGC GCC AAA C ATT CCG GGG ATC CGT CGA CC
FrwC_con_Fwd	GTG CGC AGG TTG TGC CAC TC
FrwC_con_Rev	GTC GGC GCG TTT TAC CGC GT
FraB_del_Fwd	CCT GGA TAA TGG ATT CAC CGT TGG TGG CGC ATT GCG ACT A TG TAG GCT GGA GCT GCT TCG
FraB_del_Rev	GAA AAC TGG AAG AAG AAA GGC GTA TTC GCA TCG GTA ATT T ATT CCG GGG ATC CGT CGA CC
FraB_con_Fwd	CCG TTT TTG AAT CGG TAG AC
FraB_con_Rev	TTC CGA TTC CAG GTG GCC GT
ulaA_3_del_Fwd	CAC CAT TAT CTG CAC GGC GAT ACT GGT TTC CCT GTA TG TAG GCT GGA GCT GCT TCG
ulaA_3_del_Rev	ACG CAA CCA AAG ATT TCA ATC ATC CCC ATC ACC AGA CAA AATT CCG GGG ATC CGT CGA CC

ulaA_3_con_Fwd	CCA GAT ATC GTG ATG GAA CG
ulaA_3_con_Fwd	GAG TGG TTT CAT AGG CGC TA

Table S4. qPCR primers used in this study.

Name	Sequence
Nramp1_qFd	GCAGGCCCAGTTATGGCTC
Nramp1_qRv	CAGGCTGAATGTACCCTGGTC

Table S5. Plasmids used in this study.

pKD46 Spec^{R} P_{BAD}-*gam-beta-exo oriR101 repA101*^{ts}	Datsenko and Wanner[57]	
pKD13 Carb^{R} FRT Km^{R} FRT PS1 PS4 *oriR6Kγ*	Datsenko and Wanner[57]	
pCP20 Carb^{R} CmR cI857 lPRflp oripSC101ts	Datsenko and Wanner	
pGP704 Apr; ori R6K, mob RP4, MCS of M13tgl31		Gift from Wenhan Zhu
upstream and downstream regions of *zraP* (IR715) in pGP704	This study	pNS4
upstream and downstream regions of *yfiaA* (IR715) in pGP704	This study	pNS5

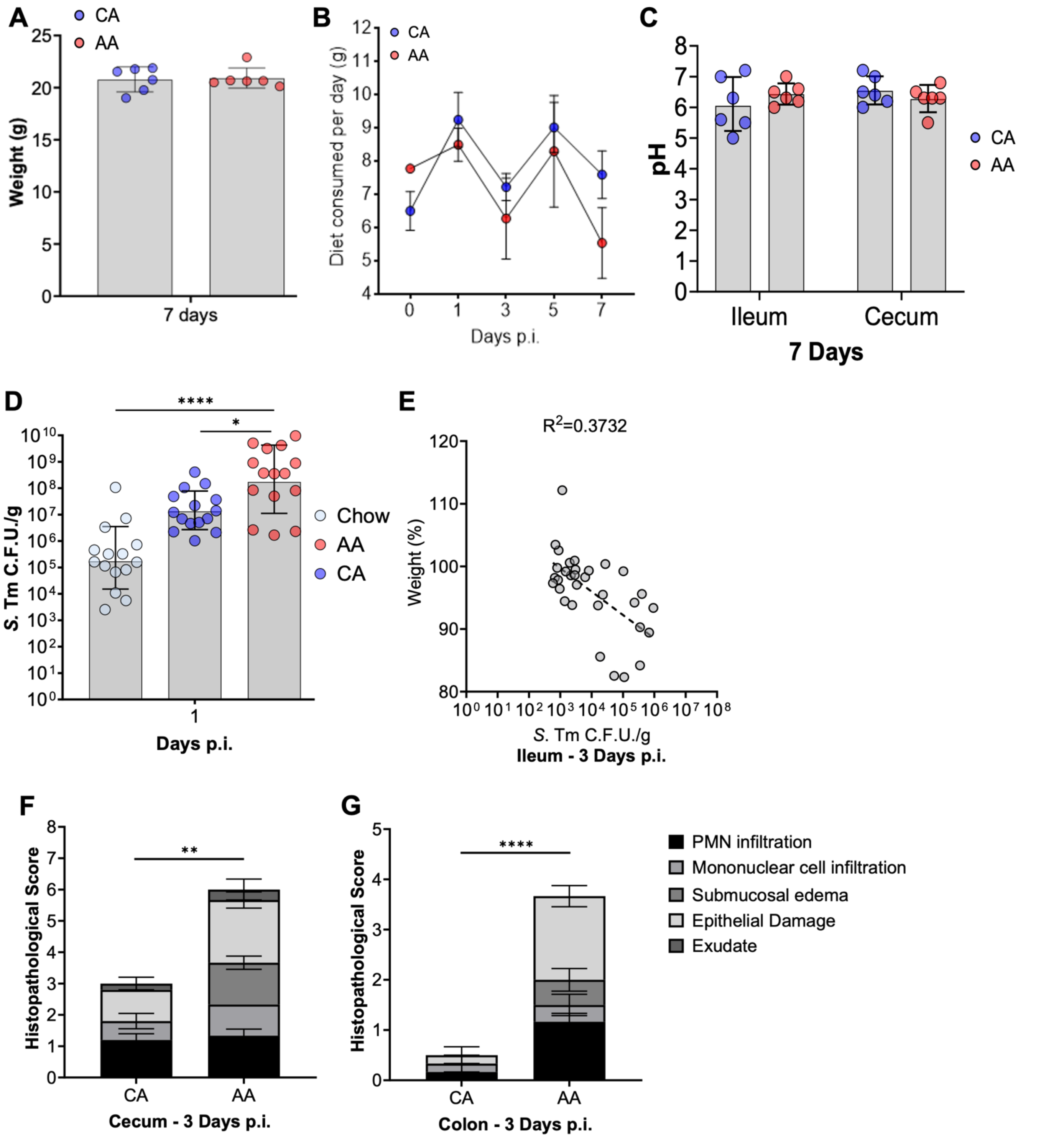

Figure S1: AA diet alone does not alter mouse weight or luminal pH of the small and large intestines. Related to Figure 1. (A-C) Naïve CBA/J mice were switched to the CA and AA diet and maintained for 7 days without infection. Every other day, food was weighed to measure the consumption rate over time. At sacrifice, mice were weighed, and intestinal content was harvested to measure pH. (D) *S.* Tm burden in feces one d.p.i. of Chow, CA, or AA diet-fed mice. Geomean and geometric SD, ANOVA, P=*,<0.05; ****,<0.0001.(E) Correlation between weight loss and bacterial burden in the terminal ileum of mice. (F-G) Histopathological scoring of cecal and proximal colon tissue three d.p.i. between CA and AA diet-fed mice. Mann Whitney, P=**,<0.01; ***,<0.001.

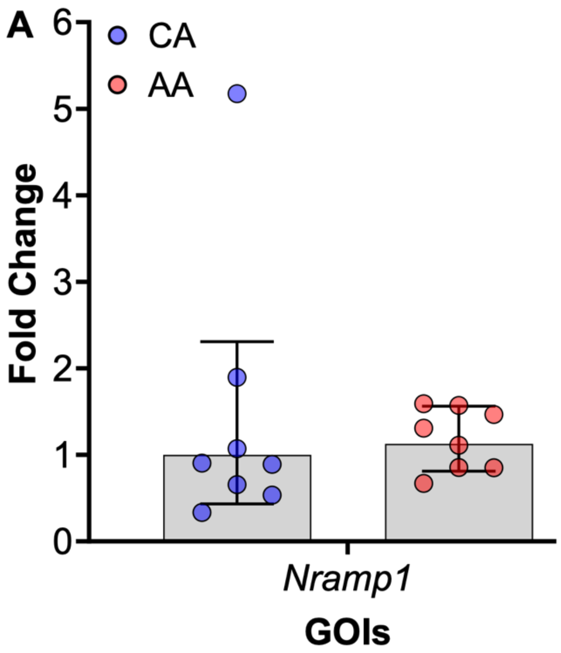

Figure S2: AA diet-fed demonstrate no differences in *Nramp1* expression 3 d.p.i. in the ileum compared to the CA diet-fed mice. Related to the Figure 2. (A) CBA/J mice were either maintained on Chow or switched to a CA or AA diet two days before infection with *S*. Tm. *Nramp1* expression in the ileum of mice measured by RT-qPCR three d.p.i.. n=8.

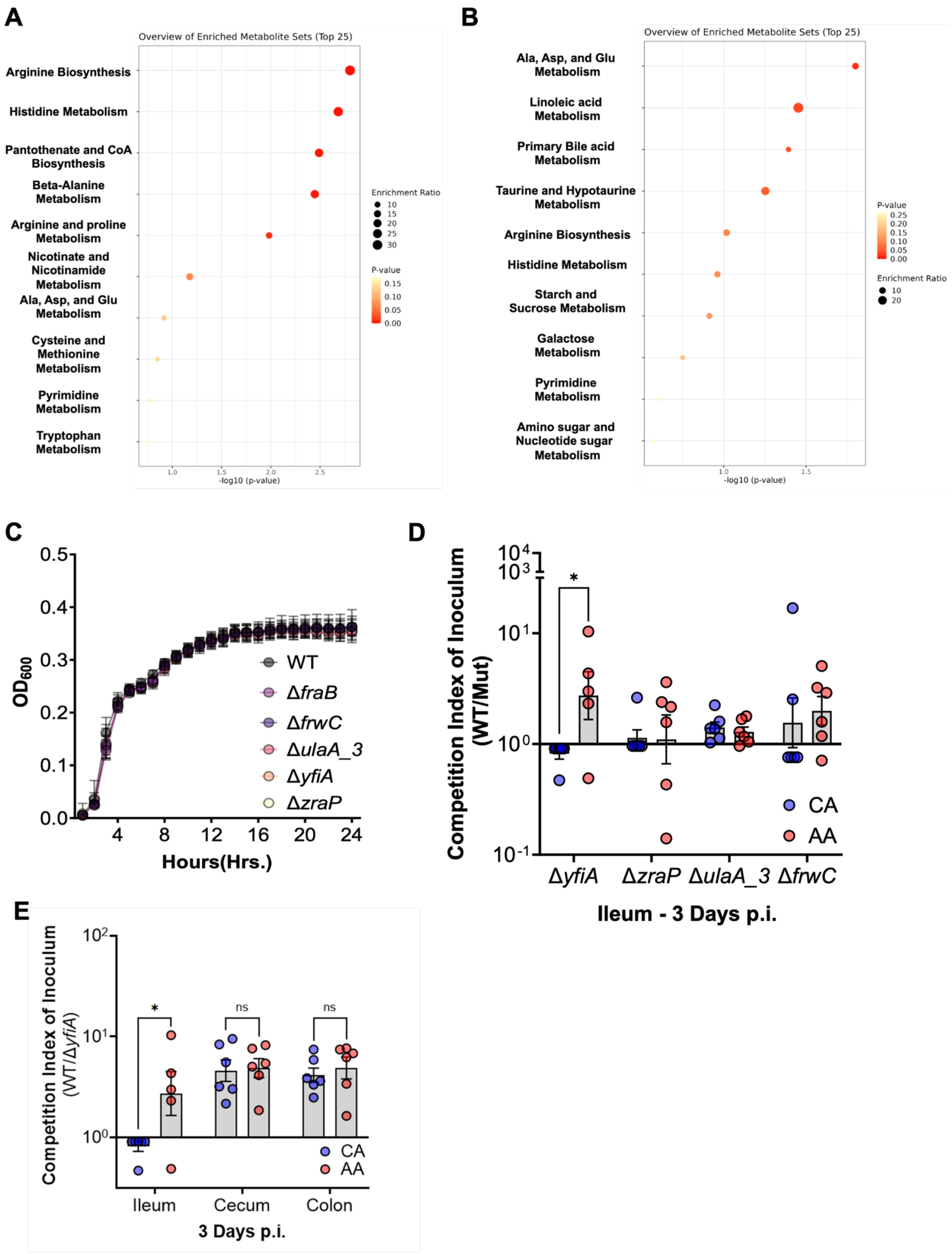

Figure S3: AA diet alters the metabolic profile of the ileum overall before infection and alters *S.* Tm metabolism *in vivo*. Related to Figure 6. Metabolites significantly altered (P<0.05 PERMANOVA; fold change greater than 2) were used for enrichment analysis via Metaboanalyst 5.0. (A) Enriched in AA-diet-fed mice; (B)

Depleted in AA-diet-fed mice. (C-D) To interrogate the relationship between our RNAseq and Metabolomics data were constructed five in-frame deletions associated with the top five DEGs or pathways within *S*. Tm. (C) Strains cultured in biological triplicate anaerobically in LB *in vitro*, measuring optical density at 600nm every hour for up 24 hours. (D) CBA/J mice pre-fed either a CA or AA diet infected with a 1:1 competitive ratio of wild type and an isogenic strain deficient in *yfiA, zraP, ulaA_3*, or *frwC;* and allowed carriage for three d.p.i.. competition index of inoculum in the ileal lumen. (E) Competitive index of mice infected with a 1:1 competitive ratio of wild type and isogenic mutants deficient in *yfiA* in the ileum, cecum, and colon content of CA- and AA diet-fed mice. N=6 per diet group. Student's t-test or Multiple t-test, P=*,<0.05.
